# Feature extraction and classification of surface discharges on an ice-covered insulator string during AC flashover using gray-level co-occurrence matrix

**DOI:** 10.1038/s41598-021-82254-9

**Published:** 2021-01-28

**Authors:** Yong Liu, Qiran Li, Boxue Du, Masoud Farzaneh

**Affiliations:** 1 Tianjin Key Laboratory of Smart Energy and Information Technology, School of Electrical and Information Engineering, Tianjin University, Tianjin, 300072 China; 2 Université du Québecà Chicoutimi, Chicoutimi, QC Canada

**Keywords:** Electrical and electronic engineering, Characterization and analytical techniques

## Abstract

This study focuses on the feature extraction and classification of surface discharges of ice-covered insulator strings during process of alternating current flashover. The test specimen was the five units suspension ceramic insulators, which was artificially accreted with wet-grown ice in the cold-climate room of CIGELE. Based on the IEEE Standard 1783/2009, flashover experiments were conducted on iced insulators to measure the minimum flashover voltage (VMF) and record the propagating process of surface discharges to flashover by using a high-speed video camera. The gray-level co-occurrence matrix (GLCM) method has been used to extract four parameters of arc discharge images features that characterize different stages of flashover process. The parameters are angular second moment (ASM), contrast (CON), inverse difference moment (IDM) and entropy (ENT). These statistical parameters of GLCM can be extracted to reveal the underlying properties of ice flashover on the insulator surface from the quantitative perspective. The different values of these indicators are representative of the different stages in the process of arc discharge. Once the value of quantitative indicators (ASM, CON, IDM, ENT) of surface discharges exceeds the threshold value, the higher flashover risk of iced insulators will appear. Hence, the proposed methods are helpful to understand and monitor surface discharge on iced outdoor insulator strings for preventing flashover accidents.

## Introduction

Ice flashover experience has been recognized as one of serious accidents for power systems operating in most of atmospheric icing regions^1–7^. From viewpoint of reducing the ice flashover accident caused by ice-covered insulators, many investigations have studied the properties of ice flashover process^8–15^. It is obtained that the icing flashover is a complex discharge phenomenon, which results from various parameters, such as the types of insulators, the shape of ice, the conductivity of the ice, amount of air gaps of iced insulator strings and atmospheric environment.

Over the past decades, many researchers have investigated the discharge mechanism and performance evaluation of iced insulators to decrease the ice flashover accident. Ma et al. used the ultraviolet photons to reveal the discharge properties of iced insulators. They found that the ultraviolet photons could be regarded as features to effectively evaluate the reliability of ice-covered insulators^16^. Wen et al. used ultraviolet (UV) imaging technology to observe the discharging phenomenon. The number of UV photons was regarded as the characteristic quantity to detect discharging, which clearly revealed a periodical corresponding relationship between the leakage current and the UV image during the icing flashover. Finally, the three-segment method of UV warning was proposed in the discharging process of ice-covered insulators^17,18^. Porkar et al. put forward the method of image charges to improve static and dynamic arc models. The improved arc model had a better correspondence with the test results than the previous model. It could be used as a powerful tool for reducing icing flashover accident of insulator strings^19^. For optimizing the method of insulator flashover, Taheri et al. established an empirical model by the electrical conductivity of the melted ice accumulation to determine the flashover voltage under icing conditions and investigated the influence of air gaps on the flashover stress of iced post insulator^20^. Liu et al. found that the arc on the long ice-covered insulators need more time up to the critical length and the development of arc will be easily effected by the decreasing of melted water conductivity and ice shedding than the short insulators^21^. Hao et al. researched the natural iced of in-service glass insulator string by image processing methods. Based on the GrabCut segmentation method, the algorithm of graphical shed spacing and graphical shed overhang are presented by recognizing the convexity defects of the contours of ice-covered insulators^22,23^.Li et al. analyzed the discharge images of ice-covered insulator during the flashover for the characteristic extraction. They use the image gray value and the arc length as the characteristic value for the flashover prediction^24^.These previous studies are helpful to improve the ability of judging the accident risk of insulator in power system. Nevertheless, these previous investigations have disadvantage dealing with the complexity phenomenon of ice accretion and indeterminacy performance of partial discharge arcs, so it is hard to exactly locate initiation and propagation of partial arcs discharge along surface of iced insulator strings, which influences the condition of discharge development and the significant factors of flashover experience^25–28^.

Up to now, although the optical measurement can record strength and dynamic distribution of surface discharges, few investigations have been conducted on the feature extraction and analysis of visual images of arc discharge along the iced outdoor insulator strings. In this paper, the multiscale geometric analysis method is presented for the feature extraction and classification of arc discharges images of iced insulator strings during AC flashover process. Due to the indeterminacy of cold outdoor environment, the natural ice-covering flashover is complex. Thus, the artificial ice-covering flashover is different from the natural ice-covering flashover in some degree. However, the image processing method proposed in this paper can overcome these differences because of the process of ice-covering flashover can be characterized by image parameters which is only related to the pixel intensity, the homogeneity of the discharge image and the local variations in gray level. In accordance with the IEEE Std. 1783/2009, all the experiments were conducted on the five-unit suspension ceramic insulators. The minimum flashover voltages were measured and dynamic discharge behaviors were recorded by using a high-speed video camera during the flashover process. Based on the image analysis of surface discharges, the quantitative indicators were proposed to reveal the discharge propagating characteristics of the flashover on iced insulator strings.

## Test setup and procedures

**Figure 1:**
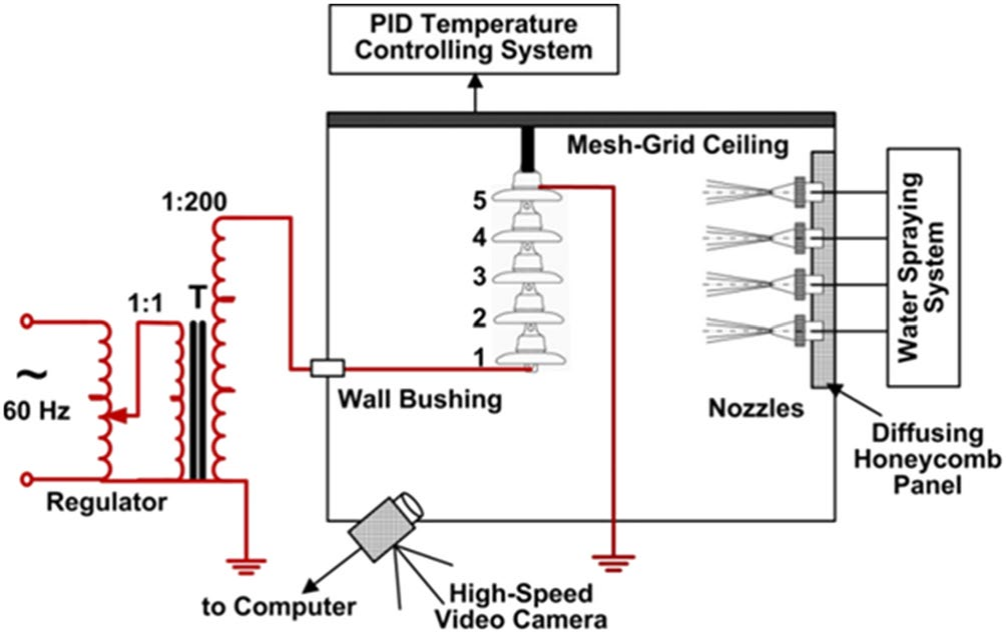
Schematic diagram of test setup at CIGELE.

Figure 1 shows a schematic diagram of test setup at CIGELE. The shed diameter, shed height, leakage distance and arcing distance of the specimen are kept at 254 mm, 146 mm, 305 mm and 809 mm. At the beginning, the deionized water is used to clean the surfaces of the insulator units.

The tests were conducted in a designed artificial climate room with a length of 4.8 m, a wide of 2.8 m and a height of 3.5 m of CIGELE Laboratories. By using the proportional integral and differential system, the freezing devices can make the ambient temperature drops to -12 °C after the test setup was fixed. When making the insulators under the setting room temperature last about sixteen hours, the voltage of 75 kVrms (15 kVrms per unit) energized on insulators. Meanwhile, the water conductivity was set at 30 µS/cm. The spray device mainly consists of a water supplying system and a wind blowing equipment. Ice was formed from super cooled droplets produced by the former system through four oscillating nozzles, which can accrete ice in the form of a uniform ice thickness on the surface of insulators; The latter system produced the uniform air flow through a series of fans with a diffusing honeycomb panel. The wind speed was fixed at 3.3 m/s to blow on the windward side of insulators in the ice accumulation period and the up-to-down oscillating rate of nozzles was 16 rpm.

Once the ice accretion was finished, then the flashover experiment was prepared and the duration of the preparation was approximately two minutes. After that, for reaching the estimated flashover voltage, the voltage was reapplied on insulators with a rate of 3.9 kV/s. Once flashover appears or no flashover for duration of 15 min, the applied voltage was diminished or increment by the settled voltage step of 3 kV for following experiment. The minimum flashover voltage (VMF) and the maximum withstand voltage (VMW) are determined when VMF and VMW differs by a voltage step of 3 kV, and when VMF is applied two flashovers in all experiments were produced. Simultaneously, the high-speed video camera which has 256 grey levels was selected visually for recording the performance of arc discharges by the rate of six thousand frames per second.

## AC flashover process of iced insulators

### Ice accretion process of insulators

**Figure 2:**
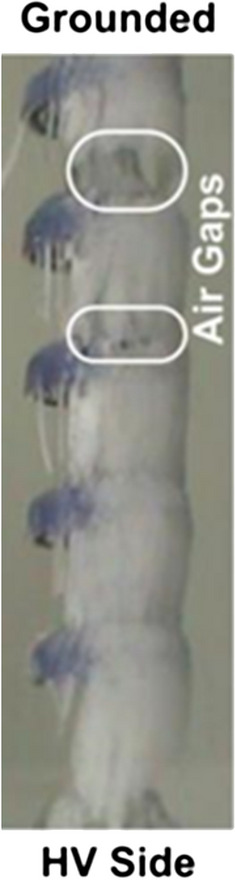
Surface performance of iced insulator strings.

Before icing flashover tests, the icing state of ice-covered insulators is shown in Fig. 2. Ice accumulation process on energized specimen is the result of the test voltage and the icing state. Because of the leakage current produced Joule heat, the test voltage can cause the melting and dripping of accreted ice, which inhabit the process of icing accumulation. The icing state have energetic influence on keeping the length and amount of icicle increased between the 2 sheds and ice layer formed on surface of insulators. When the cold room can maintain the room temperature at − 12 °C, the low-temperature droplet frozen on the surface of insulator sheds under the test condition. The state of icing regime is shown in Fig. 2.

### Flashover process of iced insulator strings

**Figure 3:**
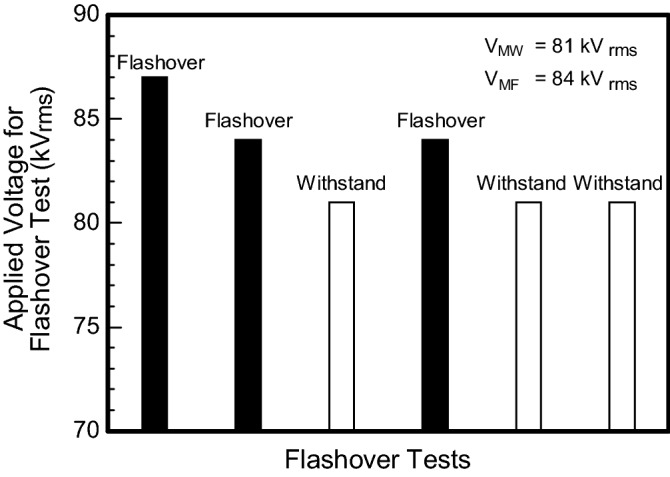
Experimental determination of maximum withstand voltage (V_MW_) and minimum flashover voltage (V_MF_) from flashover tests.

When the icing accumulation completed, the icing flashover test was carried out to obtain VMF with the value of 84 kVrms, as depicted in Fig. 3. The icing flashover phenomena is associated with the arc discharges along air gaps and residual resistance of icing layer^29,30^. Under the same condition of icing accumulation, the performance of ice accreted on insulator strings are identical, as shown in Fig. 2.

**Figure 4:**
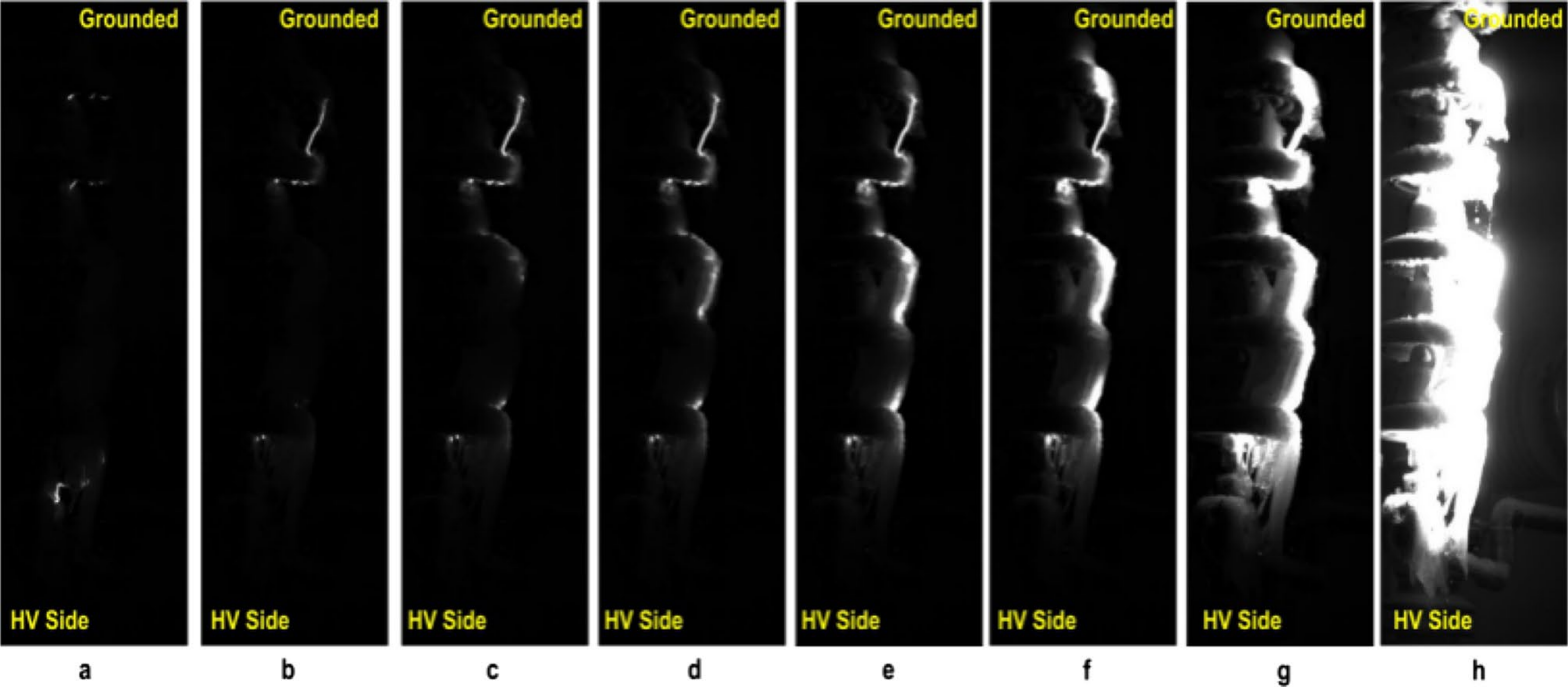
Process of arc discharges to flashover on the iced insulators: (**a**) Stage I(the initiation of test voltage), (**b**) Stage II, (**c**) Stage III, (**d**) Stage IV, (**e**) Stage V, (**f**) Stage VI, (**g**) Stage VII, (**h**) Stage VIII (the occurrence of ice flashover).Stage I and stage II are initial stage of surface discharge. Stage III to stage VII are the development stage of surface discharge. The stage VIII is stage of complete flashover.

Due to the high-speed video camera operated at the sampling rate of 6000 fps, there are a large number of repetitions of images at the same stage during the flashover process. In different flashover stages, there are obvious differences in flashover arc length, flashover arc position, continuity of flashover arc, and flashover arc area, and there will be dark-bright-darkness in each flashover stage during the experiment process. This article combines the various characteristics of the above flashover arc, divides the entire flashover process into different stages, and selects clear and obvious pictures in each stage as the research object of this article, so as to fully reveal the process from arc discharge to flashover. As shown in Fig. 4, the variation of VMF corresponds to the different discharge behaviors during ice flashover process. It can be observed that it is quite different from discharge initial, propagation to flashover.

1. Initial stage of surface discharge: Generally, due to the insulators was energized at AC voltage, ice accumulated on insulator surface is not symmetrical. Moreover, most of ice accretion along the windward side of the insulators surface under the test icing environment, and the thermal energy of discharge generated, ice shedding, all of these led to the icing-free air gaps formed on the insulators. According to observations from Fig. 4a–c, the surface discharge of ice-covered insulator strings arise from generation and extension of arc across air gap, in which the partial arcs generated from inceptive status, at a relatively slow speed due to the indeterminacy and complexity of air breakdown. The insulation properties is not extremely weaken because of the icing layer is cooling and arid. But, the electric field distribution across the ice plane was changed by the existence of high conductive ice-melted layer, which make the most of service voltage passing through air gap. Hence, the icicle ice plane gap has a corona inception behavior.
2. Development stage of surface discharge: The discharge arc spread from ice layer to the bottom side of insulator strings after that it along the icing caps under the freezing conditions^29^. The leakage current generated heating energy on ice plane and also in the ambient air, which has a positive effect on the arcs re-ignition under the applied voltage. When the applied voltage near the minimum flashover voltage level, the electrical discharge sustaining stable burning in the air gaps. During AC pollution flashover process, the resistance of polluted surface is a vital factor in extinguishing and re-ignition of arcs discharge. Similarly, the surface resistance of iced insulators play a fundamental part in arc formation, re-ignition and propagation under the applied voltage. Arcs are unable to kept in a stationary location under the AC voltage, but instead develop and extinguish at each half-cycle of AC voltage. The arc root is generated in the air gaps that led to the non-uniformity distribution of current along the surface of iced insulators, which turn to effects the residual surface resistance. If this resistance is low, the arc current will be higher. Therefore, the arcs may develop across the air gaps directly from high-voltage electrode to the grounded line.
3. Stage of complete flashover: The partial arcs across the air gaps then move to the HV electrode near by, which induce the distribution of voltage to shift on the iced insulators. The stable and bright discharge arcs extend between the icicles and electrode. The local discharge arcs can spread along the ice plane with an increasing speed. The luminosity of discharge path show longer and bigger characteristics in Fig. 4h, compared with the Fig. 4a–g. When local arcs across the most of the dry arcing distance and complete the connection of multi-arcs in air gap, it evolves into the flashover arc. Once white arc proceeds to flashover, the arc path follows the arcing distance. In such a instance, the higher energy produced by flashover may cause the insulator have a disruptive problem.

## Feature extraction and classification of discharge images using gray-level co-occurrence matrix

### Gray-level co-occurrence matrix

**Figure 5:**
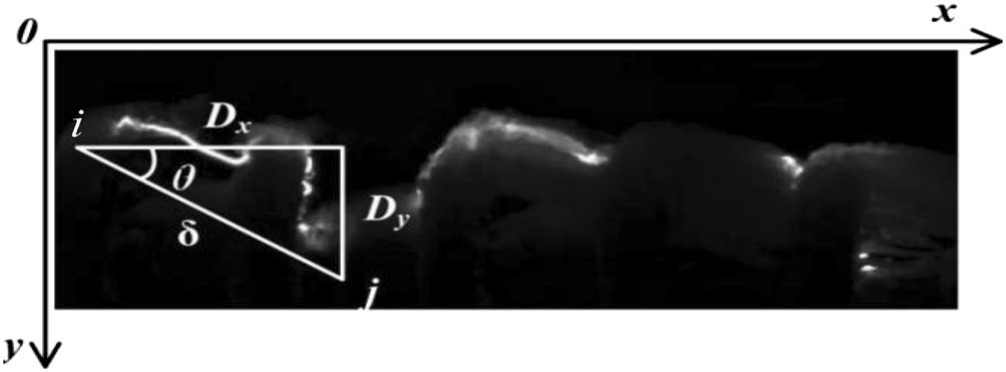
Calculation example of GLCM of surface discharge image along an ice-covered insulator string.

GRAY-level co-occurrence matrix (GLCM) with the related statistics is known as one of popular estimating method to reveal the image properties. The GLCM is defined as Eq. (1), which is a square matrix containing the relative frequency of neighboring pixel relationships of a 2-D image at the position of the corresponding gray levels of pixels separated by a certain distance in a given direction, as shown in Fig. 5.

1 $$ P\left( {i,j,\delta ,\theta } \right) = \left\{ \begin{gathered} \left( {x,y} \right)\left| f \right.\left( {x,y} \right) = i,f\left( {x + Dx,y + Dy} \right) = j; \hfill \\ x,y = 0,1,2, \cdots ,N - 1 \hfill \\ \end{gathered} \right\} $$

where P(i, j, δ, θ) denotes the value of frequency at which the pixel intensity pair i and j appear together, δ is the distance of separation between the two pixels which is often taken as the integral multiple of gray levels and θ is the direction of the two pixels which is often taken as 0°, 45°, 90° and 135°.

After obtaining the GLCM of discharge images, the statistical parameters of GLCM are extracted to reveal the underlying properties of arc discharge from the quantitative perspective. The indicators are angular second moment (ASM), contrast (CON), inverse difference moment (IDM) and entropy (ENT). Each stage of arc discharges can be indicated by the four parameters which are extracted from the selected images through gray-level co-occurrence matrix (GLCM) method. The basic principle of the GLCM method is to analyze the pixel characteristics of the image. The influence of different ice thickness, wind speed and other factors on the flashover process is finally reflected in the change of image parameters. The GLCM method proposed to process the image parameters. The change of the image parameters will only affect the calculation result, not the calculation accuracy. Therefore, factors such as different ice thickness and wind speed will not affect the performance of the GLCM method. The model is only related to the image parameters, such as the pixel intensity, the homogeneity of the discharge image and the local variations in gray level. And this model can be applied to insulators with different geometric shapes and different surface contamination levels.

ASM can be obtained as Eq. (2) to reflect the homogeneity of the discharge image. The value of ASM shows the strength of homogeneity, that is the pair correlation P(i,j) of the whole area nearly independent of the distance between i and j. So if the discharge image is homogeneous, the ASM value is smaller. While if the discharges show the area-wide uniformity in any direction, the ASM value becomes higher.

2 $$ ASM = \sum\limits_{i = 0}^{N - 1} {\sum\limits_{j = 0}^{N - 1} {\left\{ {P\left( {i,j} \right)} \right\}^{2} } } $$

CON is calculated by Eq. (3), which is a measure of the local variations in gray levels of the discharge image. The higher CON value shows that there are larger neighboring gray level differences in the image.

3 $$ CON = \sum\limits_{{\left| {i - j} \right| = 0}}^{N - 1} {\left| {i - j} \right|^{2} } \left\{ {\sum\limits_{i = 1}^{N} {\sum\limits_{j = 1}^{N} {P(i,j)} } } \right\} $$

The definition of IDM is shown as Eq. (4). Although the IDM is related to the homogeneity of the image, it is different with the ASM. Due to the weighting factor (1 + (I − j)^2^), the far separate areas (i ≠ j) can have little influence on the IDM, which means the higher IDM value for local homogeneity and lower for the local inhomogeneity. Therefore, IDM is a measure of the appearance frequency for near neighboring pairings while CON is that of distant pairings.

4 $$ IDM = \sum\limits_{i = 0}^{N - 1} {\sum\limits_{j = 0}^{N - 1} {\frac{1}{{1 + \left( {i - j} \right)^{2} }}P\left( {i,j} \right)} } $$

ENT is defined by Eq. (5)

5 $$ ENT = - \sum\limits_{i = 0}^{N - 1} {\sum\limits_{j = 0}^{N - 1} {P\left( {i,j} \right)\log \left[ {P\left( {i,j} \right)} \right]} } $$

### Quantitative indicators of surface discharge characteristics using GLCM

The variation of ASM of surface discharge images during AC flashover process is shown in Table 1. As the discharge arcs from initial status to the flashover, the value of ASM shows the decreasing tendency, and the decrease from Stage VII to Stage VIII (Flashover) is especially noticeable. Meanwhile, at each stage, the difference in ASM at different directions is small. This means the increase in the homogeneous distribution of surface discharges during the flashover process. The homogeneous area, containing only a small number of gray levels, is related to strong and condensed surface discharges. By observing the process of arc discharges to flashover (Fig. 4), the phenomenon of flashover was caused by the air-gap stable discharges and then developed by the continuous of arcing discharges corresponded to the melting of icicles. Finally, finished by connection with local arcs and the arcs elongated to reach critical conditions of AC flashover. The variation in ASM value is found well related to these surface performances, where the higher value is representative of the weak and random discharges, and the lower value corresponds to the relatively stable discharge behaviors at the ice-free regions (air gaps).

**Table 1:** Variation of ASM of surface discharge images during AC flashover process.

Stages	0°	45°	90°	135°
Stage I	0.9895	0.989	0.9895	0.989
Stage II	0.9791	0.978	0.9834	0.978
Stage III	0.9585	0.9617	0.9636	0.9536
Stage IV	0.8785	0.8825	0.9079	0.8675
Stage V	0.8159	0.8115	0.8348	0.7922
Stage VI	0.7827	0.7672	0.8107	0.7625
Stage VII	0.7229	0.7148	0.7686	0.7101
Stage VIII	0.2731	0.2462	0.3104	0.2453

Table 2 gives the variation of CON of surface discharge images during AC flashover process. CON represents the difference of the concentration in the discharge images due to the ǀi − jǀ^2^ term, which can indicate the relative location of surface discharges along the ice-covered insulator string. If the discharge image shows a large value of this parameter, it can be obtained that the concentration of surface discharges is relatively localized in some specific spatial regions and the sustainable arcing discharge channel is being formed for the occurrence of ice flashover on the insulator surface. As shown in Table 2, the CON value increases from the initiation of applied voltage (Stage I) to the flashover occurrence (Stage VIII), which can be classified into three different parts with the apparent increasing value. At Stages I to III, the insulator surface is composed of the ice layer associated with the air gaps, which results in the discharge occurring at the regions of intensified electric field. The discharges are characterized as randomness, gloominess and instability (Fig. 4a–c), having the lower valued of CON. From Stages IV to VII, the increase in CON value is connected with the development of arcing discharges along the iced insulators (Fig. 4d–g).

**Table 2:** Variation of CON of surface discharge images during AC flashover process.

Stages	0°	45°	90°	135°
Stage I	0.0053	0.0055	0.0053	0.0055
Stage II	0.0105	0.0111	0.0053	0.0111
Stage III	0.0368	0.0277	0.0263	0.0388
Stage IV	0.1737	0.1662	0.0632	0.1773
Stage V	0.5368	0.5208	0.2053	0.615
Stage VI	1.3895	1.4958	0.6158	1.5845
Stage VII	3.6053	3.6011	1.2842	4.4377
Stage VIII	7.8053	7.5623	1.4316	9.6122

The variation of IDM of surface discharge images during the development of arc discharges to the flashover is shown in Table 3. It is found that an obvious tendency of the variation of IDM to decrease during AC flashover process. It can be clearly observed that the value of IDM has a significant decrease from Stage VII to Stage VIII (Flashover), which indicates that the local distribution of surface discharges is inhomogeneous and the flashover appears on the iced insulators surface. As the Joule heat causes the ice melting, the propagation of surface discharges are influenced by conductivity of ice-melted water. The residual surface resistance will decline because the conductivity of melted ice can be higher in the progress of arc discharges. Hence, the arcs connect to the critical length, leading to the complete flashover. The variation of IDM value also reflects discharges process, and the higher value is related to the initial stage of arc discharges. While the arc discharge becomes much brighter and longer, the IDM reaches a lower value.

**Table 3:** Variation of IDM of surface discharge images during AC flashover process.

Stages	0°	45°	90°	135°
Stage I	0.9895	0.989	0.9895	0.989
Stage II	0.9791	0.978	0.9843	0.978
Stage III	0.9584	0.9617	0.9635	0.9562
Stage IV	0.8781	0.8822	0.9077	0.8669
Stage V	0.8152	0.811	0.8345	0.7914
Stage VI	0.7821	0.7666	0.8103	0.7617
Stage VII	0.7273	0.7141	0.7682	0.7094
Stage VIII	0.2698	0.2415	0.3091	0.2405

Table 4 shows the variation of ENT of surface discharge images in the process of AC flashover. The ENT value can be used to describe the randomness of the arc discharge images. The nonhomogeneous images have high ENT value, whereas homogeneous scenes have low ENT value. As shown in Table 4, the ENT value increases from the initiation of applied voltage (Stage I) to the flashover occurrence (Stage VIII). The arcs start to burn along the air gaps where the intensity of electric field is extremely high. The discharges are characterized as gloominess and weakness, which results in the lower valued of ENT from Stages I to III (Fig. 4a–c). With the development of the discharges during Stages IV to VII (Fig. 4d–g), the increase in ENT value corresponds to the phenomenon that the arcs approach the critical length and form longer discharge paths, finally develop the complete flashover at Stage VIII.

**Table 4:** Variation of ENT of surface discharge images during AC flashover process.

Stages	0°	45°	90°	135°
Stage I	0.0365	0.0382	0.0365	0.0382
Stage II	0.0657	0.0686	0.0548	0.0686
Stage III	0.1285	0.1204	0.1154	0.1342
Stage IV	0.3464	0.3452	0.2832	0.3622
Stage V	0.547	0.5721	0.4918	0.6071
Stage VI	0.7827	0.7672	0.8107	0.7625
Stage VII	0.9212	0.9624	0.785	0.978
Stage VIII	2.2671	2.3593	2.0339	2.3798

**Figure 6:**
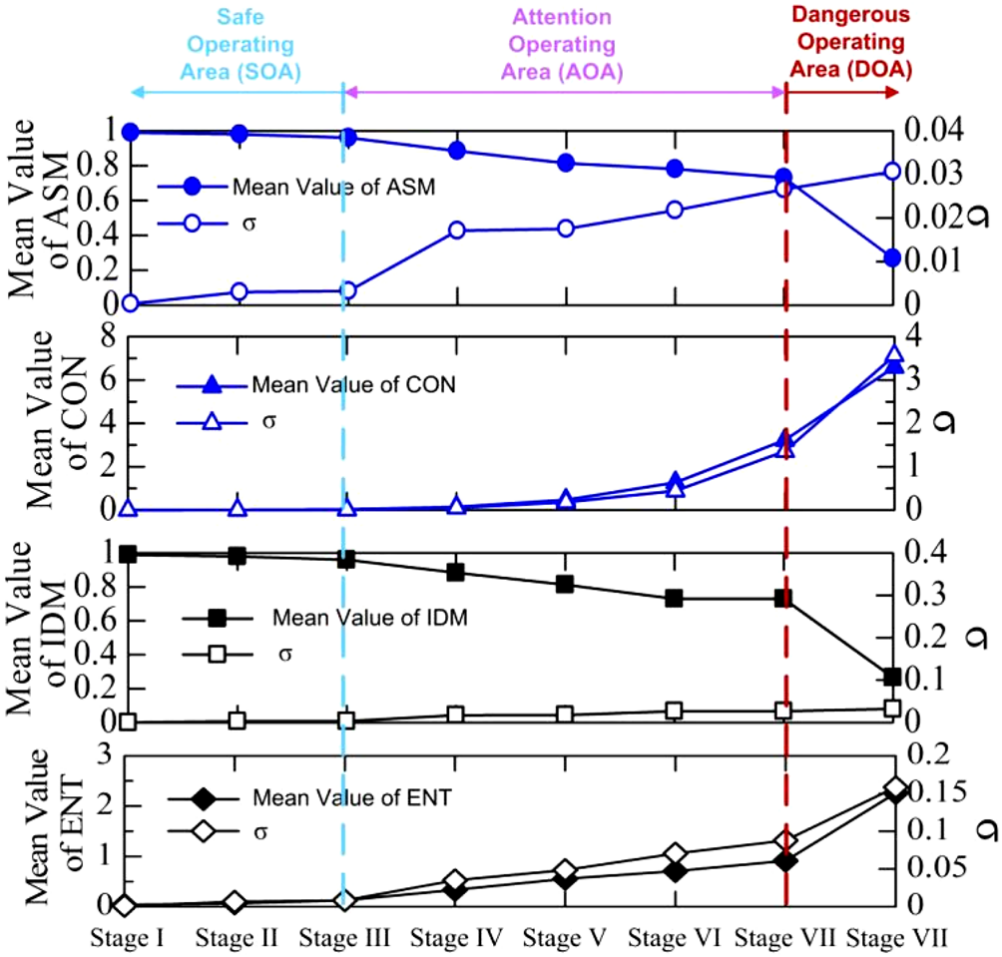
Mean value of quantitative indicators (ASM, CON, IDM, ENT) of arc discharges during the flashover process of iced insulators.

Figure 6 shows the variation of mean value of quantitative indicators (ASM, CON, IDM, ENT) of arc discharges images in the AC flashover process. Starting from comparative analysis of the Stages I to VIII calculation results, the standard deviation of results is less than 4%, which means that the dispersion of value of indicators is slight. From Stages I to III, the subtle changes in discharge image data correspond to the gloomy and weak discharge behaviors. At the stage of discharge propagation, the variation value of indicators in accordance with local arcs enlarge and the increment of local arcs. Due to the sustained burning of white arcs expanded across icing layer stability to reach the arc critical length, the value of indicators shows an inflection point during the stage of flashover, which can predict the higher risk of flashover appearance on the iced insulator strings.

## Conclusion

According to investigate the statistical indicators of GLCM, the arc discharge properties of icing flashover process are analyzed. The significant conclusions are obtained as follows:

1. The reason of the appearance of local arcs is the insulators are bridged by icicles that led to the nonuniform distribution of electric field. As stronger white arcs generated, they extend across the ice plane until reach the critical length with increasing the applied voltage.
2. The melting water conductivity increases, which causes the increase of arc discharge intensity. The residual icing layer cannot withstand the applied voltage on it and flashover will take place on the surface ice.
3. These four statistical parameters of GLCM can be extracted to reveal the discharge mechanism of ice flashover process. The different values of these indicators correspond well to the different stages of arc discharge process, which can be used as the risk value for monitoring iced insulators flashover. The proposed model is only related to the image parameters, such as the pixel intensity, the homogeneity of the discharge image and the local variations in gray level. Therefore, this method can be applied to the different insulator geometries and surface contamination levels.
4. The higher ASM value is representative of the weak and random discharges, while the lower ASM value corresponds to the relatively stable discharge behaviors at the air gaps. When the value of ASM exceeds 0.9, the stage of arc discharge is in safe operating area (SOA). When the value of ASM is within 0.9–0.4, the stage of arc discharge is in attention operating area (AOA). When the value of ASM is less than 0.4, the stage of arc discharge is in dangerous operating area (DOA).
5. The value of CON is a measure of the local variations in gray levels of the discharge image which can indicate the relative location of surface discharges along the ice-covered insulator string by analyzing, whereas the IDM is a measure of the appearance frequency for near neighboring pairings. When the value of CON is below 0.1, the stage of arc discharge is in safe operating area (SOA). When the value of CON is between 0.1 and 6.0, the stage of arc discharge is in attention operating area (AOA). When the value of CON exceeds 6.0, the stage of arc discharge is in dangerous operating area (DOA).When the value of IDM exceeds 0.9, the stage of arc discharge is in safe operating area (SOA). When the value of IDM is within 0.9–0.3, the stage of arc discharge is in attention operating area (AOA). When the value of IDM is below 0.3, the stage of arc discharge is in dangerous operating area (DOA).
6. The value of ENT can be used to describe the randomness of the arc discharge images. The nonhomogeneous images have high ENT value, while homogeneous scenes have low ENT value. When the value of ENT is less than 0.2, the stage of arc discharge is in safe operating area (SOA). When the value of ENT is between 0.2 and 2.0, the stage of arc discharge is in attention operating area (AOA). When the value of ENT exceeds 2.0, the stage of arc discharge is in dangerous operating area (DOA).
